# Chronic Kidney Disease, Obesity, and Hypertension: The Role of Leptin and Adiponectin

**DOI:** 10.1155/2012/943605

**Published:** 2012-12-23

**Authors:** M. Tesauro, A. Mascali, O. Franzese, S. Cipriani, C. Cardillo, N. Di Daniele

**Affiliations:** ^1^Division of Internal Medicine, Department of Medicine of the Systems, University of Rome “Tor Vergata”, Rome, Italy; ^2^Division of Nephrology, Department of Medicine of the Systems, University of Rome “Tor Vergata”, Rome, Italy; ^3^Division of Pharmacology, Department of Medicine of the Systems, University of Rome “Tor Vergata”, Rome, Italy; ^4^Department of Internal Medicine, “Università Cattolica del Sacro Cuore”, Rome, Italy

## Abstract

Chronic kidney disease is a major public health problem and characterized by a progressive loss in renal function over a period of months or years as defined by structural or functional abnormalities of the kidney. Several elements contribute to determine a progression of the kidney injury, inducing a worsening of renal damage and accelerating the decline of renal function: obesity and hypertension are two known factors of kidney progression. Remarkable improvements have been recently achieved in the study of the endocrine features of the adipose tissue and have been able to produce hormone-like peptides named adipokines or adipocytokines. Among these adipocytokines, which represent a link between obesity, hypertension, and chronic nephropathy, leptins and adiponectin appear to play an important role. Leptin not only is a prohypertension element (renal progression factor) through the activation sympathetic nervous, but also is able to induce prosclerotic effects directly on the kidney. In contrast, a decline of adiponectin levels has been shown to be related to a picture of hypertension: an endothelial dysfunction has been described as the main pathogenic mechanism responsible for this phenomenon.

## 1. Introduction

Chronic kidney disease is a major public health problem and characterized by a progressive loss in renal function over a period of months or years as defined by structural or functional abnormalities of the kidney. Identification and staging of chronic kidney disease are evaluated by glomerular filtration rate (GFR) and proteinuria [1].

The most important international records indicate a dramatic increase in the number of patients dependent on chronic dialysis therapy (stage 5 of chronic kidney disease) [2]. 

Several elements contribute to the onset and progression of chronic renal damage, including susceptibility, initiation, and progression factors.

Susceptibility elements are those situations conferring an increased risk of renal damage (age, familiarity, reduced nephron mass, low birth weight, disadvantaged social condition). Initiation elements are those factors able to determine kidney injury, especially in high-risk patients: diabetes mellitus, hypertension, autoimmune diseases, systemic infections, recurrent urinary infections, calculi/urinary tract obstruction, or drugs. 

Progression elements contribute to determine a progression of the kidney injury, inducing a worsening of renal damage and accelerating the decline of renal function: (protein loss, hypertension, inadequate glycemic control of diabetic patients, cigarette smoke).

From a histological point of view, progression of renal damage is characterized mainly by glomerulosclerosis, interstitial leukocyte infiltration, and tubulointerstitial fibrosis [3, 4]. 

The link between obesity and chronic nephropathy has been initially set in the so-called theory of hyperfiltration. Nevertheless, remarkable improvements have been recently achieved in the study of the endocrine features of the adipose tissue, able to produce hormone-like peptides, named adipokines or adipocytokines. Among these adipocytokines, which represent a link between obesity, hypertension, and chronic nephropathy, leptins and adiponectin appear to play an important role: leptin not only is a prohypertension element (renal progression factor) through the activation sympathetic nervous, but also is able to induce prosclerotic effects directly on the kidney. 

In contrast, a decline of adiponectin levels has been shown to be related to a picture of hypertension: an endothelial dysfunction has been described as the main pathogenic mechanism responsible for this phenomenon. 

## 2. From Hyperfiltration to the Endocrine Role of Adipose Tissue

The theory of hyperfiltration was introduced in the last century in order to elucidate the evolution of chronic kidney disease towards the end-stage renal disease, regardless of persistence or resolution of the cause. This theory is based on the hypothesis that a critical reduction of the number of functioning nephrons would lead to altered functional and structural adaptations. Initially renal functions may be maintained, but in the long term a progressive glomerulosclerosis, proteinuria, and renal failure can be observed [5, 6]. As a result, hemodynamic adaptations in the surviving glomeruli can be described, including vasodilation of afferent glomerular arterioles, increased filtration fraction, and increased glomerular transcapillary hydraulic pressure. 

Still, these theoretical principles do not get easy application to clinical practice: a significant number of patients showing severe renal mass reduction (i.e., patients with bilateral renal carcinomas submitted to >80% renal parenchyma resection) did not develop proteinuria or renal insufficiency in the course of followup [7]. 

Hyperfiltration theory has been suggested to be the cause of pandemic obesity and type 2 diabetes. As frequently demonstrated, most obese and diabetic individuals tend to show the same pattern of glomerular hemodynamics as compared to patients and animals with reduced renal mass (preglomerular vasodilation, increased glomerular filtration rate, and filtration fraction). Glomerular filtration rate (GFR) has been shown to be higher in obese individuals, while proteinuria and secondary glomerulosclerosis are now recognized as specific complications of severe obesity [3]. 

Huge progress has been recently achieved in the study of the endocrine features of adipose tissue, which is able to produce several hormone-like peptides named grouped adipokines. Adipose tissue contains mainly adipocytes, but also preadipocytes (not yet loaded with lipids), endothelial cells, fibroblasts, and leukocytes.

Therefore, besides its function as an energy store, a new important role is emerging for adipose tissue in the control of many pathological processes. 

Several products from adipose tissue have been isolated, including adipocytokines. 

The term adipocytokine is used to describe cytokines mainly produced by adipose tissue, although adipocytokines are not exclusively derived from this compartment. An important link has been shown between adiponectin, leptin, resistin and visfatin and obesity, insulin resistance, and related inflammatory disorders [8]. 

Most abundant adipocytokines secreted by adipocytes include adiponectin and leptin, together with nonadipocytokine immunological mediators, such as tumour- necrosis factor (TNF), interleukin-6 (IL-6), IL-1, and CC-chemokine ligand 2 (CCL2, also known as MCP1); mediators of the clotting process, such as plasminogen- activator inhibitor type 1; and certain complement factors providing an important link between the immune and metabolic systems.

## 3. Evidences and Effects of Increased Sympathetic Activity in the Obese Patient

Fat present around abdominal viscera in mesentery and omentum, known as visceral fat, is different from that present in subcutaneous areas (subcutaneous fat). Visceral fat induces Sympathetic Neural Activation, while subcutaneous obesity is not associated with this phenomenon. However, among obese individuals, sympathetic activation occurs almost only in those with high arterial pressure [9, 10]. Noteworthy, Indian Pima population, characterized by obesity and hyperinsulinemia, shows low prevalence of hypertension and, in parallel, low signs of sympathetic neural activation [11]. 

Several evidences show an amplified sympathetic neural activation in obese patients. 

Obese patients show higher serum catecholamine levels as compared to normal individuals, together with elevated noradrenaline output [12, 13]. 

Analysis of muscle sympathetic nerve activity, through the study of peroneal nerve in obese patients showing high visceral fat level, demonstrated higher “firing” frequence as compared to normal individuals [14]. Moreover, Grassi et al. [15] evidenced a decreased sympathetic activity (through muscle sympathetic nerve activity record) in obese individuals after 16 weeks of hypocaloric diet. 

**Figure 1:**
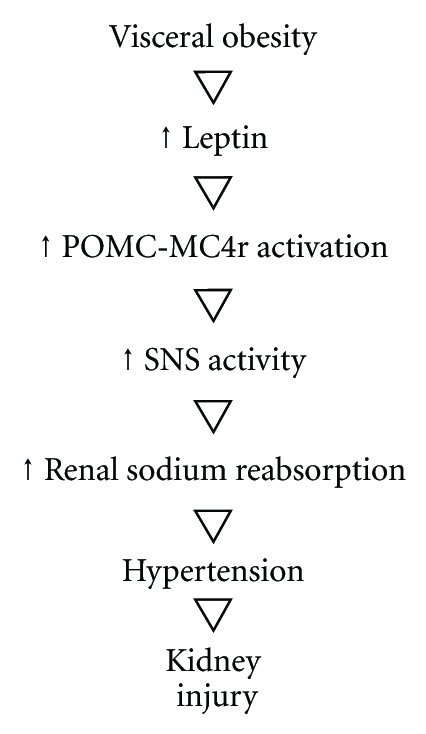
Leptin and SNS activity.

An increased activity of SNS could be related to upregulated renal sodium reabsorption. It can be suggested that obese children are deficient in their natriuretic response to an increased blood pressure [16]. In normal blood pressure individuals, a transitory increase in the systemic blood pressure induces higher levels of sodium and water excretion, mainly as a consequence of a reduced reabsorbance at the ascending limbo of Henle in the outer medulla. This phenomenon, known as pressure-related natriuresis, leads to a decreased effective circulating volume [17, 18], Figure 1.

These observations set the basis for the identification of a procedure of percutaneous renal sympathetic denervation (RDN) in individuals showing metabolic syndrome and polytherapy-resistant hypertension. Although this procedure has provided encouraging results, its long-term effects must still be evaluated. 

## 4. Leptin

Leptin is a adipocytokine belonging to class 1 cytokine superfamily and is abundantly produced by the adipose tissue. Leptin, mainly produced by visceral, subcutaneous, and pericardial adipose compartments, is also released by heart, placenta, and stomach. 

Leptin receptor, a transmembrane single-chain protein, exists in 6 isoforms with a common extracellular domain and a variable length cytoplasmic portion, codified as Ob-Ra to Ob-Rf [19], where Ob-Re represents the soluble form of the receptor. 

Most biological effects of leptin are mediated by the Ob-Rb receptor, which is highly expressed at hypothalamic level. The effects of leptin on hypothalamus include the stimulation of neuropeptides regulating the balance between appetite and energetic intake, inducing satiety and increased energy consumption. 

Leptin is able to activate POMC (proopiomelanocortin) expressing neurons in the curved hypothalamic nucleus, stimulating both production and release of alfa-MSH, which binds to MC3/MC4-R receptors expressed on hypothalamic nuclei, inducing a reduction of appetite and an increase of energy consumption [20–22]. 

Da Silva et al. showed that a prolonged MC3/MC4 receptor antagonism is able to contrast leptin stimulation of SNV [23], leading to a decrease in blood pressure values. 

Leptin receptors have also been isolated on endothelial [24] and smooth muscle cells (SMC) [25]. These observations suggest a role for this adipocytokine in controlling proliferation and migration of SMC and endothelial cells, thus influencing blood vessel tone and thickness of the wall. Besides, leptin has been shown to induce oxidative stress in endothelial cells [26]. 

It has been described how leptin levels decline during abstinence from food and increase following a number of days of hyperalimentation, when it plays its role in regulating the energetic balance. 

A “resistance” mechanism has been suggested by the observation that obese patients do not show appetite reduction and increase of energy consumption in the presence of high levels of leptin [27]. This resistence to leptin does not involve all receptors, suggesting a mechanism of selective resistence, limited to the metabolic compartment and not involving other targets of the molecule, in particular ANS and kidney [27]. 

**Figure 2:**
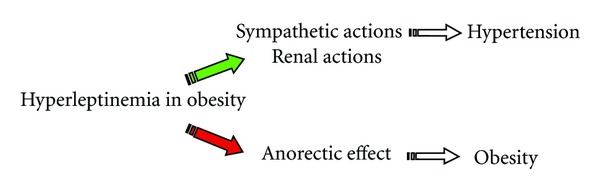
Leptin resistence.

A plausible explanation for these observations could be a decline in the passage of leptin through the hematoencephalic barrier, resulting in the lack of the anorectic effect [28] Figure 2.

### 4.1. Leptin and Activation of Sympathetic Nervous System

In different experimental models, chronic hyperleptinemia has been shown to increase blood pressure. This observation could be the consequence of the activation of sympathetic nervous system as well as the impairment of natriuresis and nitric oxide inhibition [29]. 

Shek et al. demonstrated that a chronic infusion of leptin in mice was able to induce a persistent rise in blood pressure, which returned to normal values following the suspension of the infusion [30]. 

Aizawa-Abe et al. [31] showed an increase of about 20 mmHg in systolic pressure in leptin hyperexpressing transgenic mice showing elevated urinary catecholamine excretion. 

Moreover, da Silva et al. evidenced that obesity is associated with an increased sympathetic activity, in particular in the renal compartment, while the critical role of renal innervation was demonstrated in dogs through bilateral denervation techniques [32]. 

Prior et al. were able to identify a close relationship between plasmatic leptin concentration and renal sympathetic activity [33]. The critical role for leptin in obesity-linked hypertension has also been demonstrated by Rahmouni et al. In this study, conducted in 3 murine models resembling human Bardet-Biedle syndrome, leptin was able to induce upregulation of renal sympathetic activity and increased blood pressure values in 2 models out of 3, while blood pressure returned to normal values following gangliar block [34].

### 4.2. Leptin and Kidney Damage

The effects leptin is potentially able to exert on the kidney may contribute to the worsening of the renal function. Studies conducted in murine models have shown how hyperexpression of lectin increases the activity of sympathetic nervous system, leading to an upregulation of blood pressure and an increase of urinary catecholamine excretion [31, 35]. A small amount of the isoform Ob-RB receptor is expressed on the kidney, which on the contrary expresses a large amount of the shorter Ob-Ra isoform [36]. Leptin has been shown to induce the growth of cultured glomerular endothelial cells and increase the production of transforming-growth-factor-(TGF-) beta 1. 

No much information is available about the influence of leptin on the podocyte. A 72 h infusion of recombinant leptin is able to induce TGF- alfa 1 expression and increases the total number of proliferating cells, while a three-week infusion is associated with an amplified glomerular expression of type IV collagen [37]. Raised levels of blood pressure were not evidenced, while a significant increase in urinary protein excretion was experienced. 

Leptin induces the synthesis of type 1 collagen in mesangial cells, as well as type 4 collagen in glomerular endothelial cells contributing to extracellular matrix deposition, glomerulosclerosis, and proteinuria [38]. 

A link has been described between the increased incidence of glomerulosclerosis in patients with severe obesity and serum leptin concentration. 

Usually, obese patients show increased risk of developing glomerulosclerosis. In particular, proteinuria and chronic kidney disease can be observed following unilateral nephrectomy [39]. 

92% of the obese patients with body mass indexes (BMIs) greater than 30 developed proteinuria and renal insufficiency as compared with only 12% of those patients with BMIs less than 30 [40]. 

It has been suggested that an increase of extracellular matrix production can also be mediated by a cooperation of leptin with other soluble mediators in order to promote diabetic nephropathy. High serum levels of leptin have been described in obese hyperinsulinemic type 2 diabetic patients. 

In Pima Indians, a correlation has been described between increased urine leptin levels and augmented albuminuria, while a negative correlation was established between high urine leptin levels and GFR [41]. Patients affected by type 2 diabetes showed a link between higher serum leptin concentrations and increased urine albumin excretion. Moreover, in a model of type 2 diabetic mice, the db/db, the development of glomerular hypertrophy, glomerulosclerosis, renal insufficiency, and eventually proteinuria have been described. Noteworthy, it has been shown how neutralization of TGF-beta by specific monoclonal antibody is able to prevent the expansion of mesangial renal and renal insufficiency. Moreover, while a mesangial expansion has been observed in the hyperleptinemic db/db mice,renal disease is only rarely observed in ob/obmice, who are simply leptin deficient.

## 5. Adiponectin

Adiponectin is a 30 kDa protein mainly secreted by adipocytes. It has been described how obesity is associated to hypoadiponectinemia, probably through inhibition of gene transcription and a reduced release of the factor [42]. 

### 5.1. Adiponectin and Hypertension

Several clinical studies have established a relationship between adiponectin plasma concentration and hypertension. 

Adiponectin plasma levels have been shown to be significantly lower in patients affected by essential hypertension as compared with individuals with normal blood pressure [43]. An inverse correlation has been observed between mean-diastolic pressure and adiponectin concentration. 

Hypoadiponectinemia is a risk factor for arterial hypertension, independently from a potential condition of insulin resistance and diabetes [44]. 

Chow et al. [45] showed a correlation between a decline of adiponectin concentration and the risk of developing hypertension in normal individuals over a period of 5 years. 

Studies on adiponectin genetic variants have provided several information about the link between hypertension and adiponectin. 

The polymorphism 164 in the gene encoding for adiponectin has been associated with hypoadiponectinemia and arterial hypertension in Japanese individuals [44]. 

Experimental studies support this role for adiponectin in regulating arterial blood pressure [46]: adiponectin knockout mice do not show hypertension signs in the absence of stress inducing factors, while showing higher expression values as compared with wild type mice following a low-sodium content diet. 

In uninephrectomy lineages subjected to infusion with aldosterone, adiponectin knockout subtypes developed higher arterial pressure values as compared with wild types [47] while showing a more severe diastolic cardiac dysfunction.

### 5.2. Adiponectin and Endothelial Function

Endotelial dysfunction is a major predisposing factor for vasculopathy and is strictly associated with compliances related to obesity such as insulin resistance and arterial hypertension [48]. 

Normal levels of adiponectin seem to be fundamental for a physiological endothelial function; adiponectin plasma levels are strictly related to the vasodilatatory response. Hypoadiponectinemia has been associated with a decline of brachial arterial vasodilatatory response in diabetic patients [49]. Similarly, adiponectin knockout mice show a reduced vasodilatatory response to acetylcholine as compared with controls [50]. 

The endothelial enzyme nitric oxide synthase (eNOS) and NO control the vascular homeostasis and, in particular, the endothelial function [51, 52]. 

Adiponectin has been suggested to modulate the endogenous production of NO by endothelial cells. 

Adiponectin knockout mice show lower amount of eNOS at the level of aortic endothelium and lower plasma NO metabolite levels [46], together with higher arterial pressure levels as compared with controls. 

Nishimura et al. [53] evidenced a more severe ischemia-reperfusion-related damage in knock out mice, together with a decline of eNOS levels in the encephalon. 

In vitro studies have confirmed the role of adiponectin in regulating eNOS activity and NO production. Adiponectin stimulates phosphorylation of eNOS at serine 1177 in human endothelial cells, through the activation of kinases [54], therefore inducing an NO production [55]. 

Activation of the kinase cascade follows a possible interaction between adiponectin and AdipoR1- and Adipo R2-specific receptors [56]. 

Adiponectin also plays a role in stimulating the endothelial cyclooxygenase 2 (cox-2) with production of PGI_2_, showing vasodilatatory action. Interaction between adiponectin and endothelial cells has been shown to be mediated by the complex calreticulin/CD91. 

Therefore, adiponectin plays an important role in regulating endothelial function through the stimulation of eNOS as well as PGI_2_.

### 5.3. Adiponectin and Kidney

Several clinical studies have also demonstrated a link between hypoadiponectinemia and microalbuminuria in patients showing hypertension, as well as Japanese [57] and Afro-American obese patients [58].

Sharma et al. evidenced [59] the highest basal levels of microalbuminuria in adiponectin knockout mice, together with an alteration of podocyte pediculi. 

A recent study by Kacso et al. [60], conducted on 86 type 2 diabetic patients, with a GFR > 30 ML/MIN, has demonstrated a more evident progression of renal damage in patients characterized by hypoadiponectinemia as compared with controls.

## 6. Conclusions

Characterization of the endocrine features of fat tissue has radically modified the physiopathological view of related syndromes such as obesity, arterial hypertension and chronic renal disease. Among adipocytokines specifically produced by fat tissue, leptin and adipokine play a central role in the genesis of hypertension and renal damage. Leptins represent a crucial cause of hypertension and are important initiation and progression factors for chronic renal failure, through abnormal sympathetic neural stimulation, showing a direct prothrombotic effect on the kidney. Adiponectinemia is associated with hypertension and endothelial disfunction. 

Further studies are necessary in order to elucidate the mechanisms involved in adipocytokines-mediated role in chronic kidney disease.
